# Exercising for the Pleasure and for the Pain of It: The Implications of Different Forms of Hedonistic Thinking in Theories of Physical Activity Behavior

**DOI:** 10.3389/fpsyg.2016.00843

**Published:** 2016-06-10

**Authors:** Stephen L. Murphy, Daniel L. Eaves

**Affiliations:** ^1^Sport and Exercise Science Section, Teesside UniversityMiddlesbrough, UK; ^2^School of Sport, Exercise and Health Sciences, Loughborough UniversityLoughborough, UK

**Keywords:** hedonism, hedonic theory, pleasure-pain principle, emotion regulation, affect, exercise behavior, persuasion, emotional reasons for action

“They [pleasure and pain] govern us in all we do, in all we say, in all we think” (Bentham, 1780, p.1)

Physical inactivity is one of the most widely reported risk-factors associated with many non-communicable diseases, including cardiovascular disease, diabetes mellitus, cancer, obesity, hypertension, stroke, osteoporosis, and depression (Warburton et al., 2006; Bravata et al., 2007), as well as overall global mortality (World Health Organization, 2010). Interventions that increase physical activity can reliably reduce the impact of these undesired outcomes. However, the key question facing research and theory is how best to conceptualize the associated psychological factors, which ultimately determine adherence to behavioral change. Social-Cognitive models of behavior, including the Theory of Planned Behavior, Transtheoretical Model, and Social-Cognitive Theory, represent popular explanatory frameworks, which can help practitioners understand how best to improve physical activity behavior.

In recent years, however, the usefulness of these models has been questioned. Research shows social-cognitive models typically only account for approximately 25% of the variation in exercise and physical activity behavior, which limits the effectiveness of related interventions (Ekkekakis and Dafermos, 2012). Instead, exercise behavior may be better understood by looking beyond the purely cognitive and social components. Recent research demonstrates affective components can predict physical activity behavior. For example, Williams et al. (2008) found affective responses to acute moderate-intensity exercise predicted subsequent physical activity levels in sedentary adults between 6 and 12 months later. These findings, which have been replicated (e.g., Kwan and Bryan, 2010), suggest interventions that manipulate the affective response to physical activity could improve long-term physical activity behavior.

A growing body of literature now promotes affective models, and their synthesis with social-cognitive theories (Kiviniemi et al., 2007; Calitri et al., 2009; Lawton et al., 2009). Within some of these accounts (Williams et al., 2008; Nasuti and Rhodes, 2013), the term “hedonism,” which refers to a theory of human motivation in which individuals are motivated to experience pleasure and avoid pain, is being increasingly used to explain the predictive utility of these affective components (Ekkekakis and Dafermos, 2012). For instance, Williams et al. (2008) argues for “hedonic theory to [focus] on affective responses to behavior” (p. 232), while Cabanac (2006) asks whether the “…hedonic dimension of perception is what motivates and optimizes behavior…” (p. 111). Indeed, Ekkekakis and Dafermos (2012) declare “the foundation has now been laid for the development of a hedonic theory of exercise behavior” (p. 325). Despite this trend, there is a general failure in both the physical activity and psychology literature (in the round), to define what is *actually* meant when referencing to hedonism *per se*. Thus, the aim of this article is to first discuss the definitional problems associated with hedonism, and then briefly explore how the lack of clarity therein has hindered the development of modern affective accounts of adherence to physical activity. We hope this discourse will ultimately aid theorists and practitioners alike who seek to understand adherence better, in terms of the related affective states.

In psychology hedonism typically refers to the assumption that an individual will normally “…approach pleasure and avoid pain…” (Higgins, 1997, p. 1280), or when “…pleasure seeking is the *main* motivator of human behavior” (emphasis added, Veenhoven, 2003, p. 437). At first glance, these definitions indicate that while both the pursuit of pain and avoidance of pleasure are important drivers of behavior, other motivators can legitimately contribute their “votes” toward biasing subsequent actions. Upon closer inspection, however, a more extreme version of hedonism also exists in the literature. For instance, Sober and Wilson (1999) argue that in psychological hedonism “avoiding pain and attaining pleasure are the only ultimate motives that people have; everything else that we want, we want solely as a means to achieving those twin ends” (p. 2). Similarly, Vroom (1964) suggested hedonism's central assumption is that: “In every situation people select from alternative possibilities the course of action which they think will maximize their pleasure and minimize their pain” (p. 9). Finally, Mees and Schmitt (2008) argued that the principle postulate of classical hedonistic motivational theory is that “actions are ultimately carried out to maximize the level of one's own pleasure and to minimize one's own pain” (p. 157).

Hedonism can thus be defined in a most extreme sense, in which no other motivational drivers exist beyond *the pursuit of pleasure and avoidance of pain* [hereon referred to as Ultimate Psychological Hedonism (UPH); (Mees and Schmitt, 2008)]. Therefore, when developing modern theories of motivation for physical activity it is crucial to delineate between UPH and its less extreme counterpart [Partial Psychological Hedonism (PPH)], which can accommodate alternative motivational drivers. Indeed, such confusion is evident in Weiner's (1992) work, where hedonism is defined as “a utilitarian doctrine [which] asserts that pleasure and happiness are the chief goals in life” (p. 29). Since this statement invites the possibility of other motivational drivers, which sub-serve this “chief” goal, it is unclear if this definition invokes either UPH or PPH. In contrast, Weiner (1992) later states that “the single principle on which most theories of motivation agree is that persons seek to maximize pleasure and to minimize pain, with motivation derived from this *fundamental law*” (emphasis added, p. 353). Following his brief critique of hedonism's validity as a theory of human motivation, Weiner (1992) also states “If humans do not always act as hedonic maximizers, then other motivational principles are needed” (p. 357). Moreover, when Higgins (1997) states: hedonism refers to the idea that individuals “approach pleasure and avoid pain” (p. 1280), and that hedonism “…is the basic motivational assumption across all areas of psychology…” (p. 1280), it appears easy to conflate these two forms of hedonism.

Thus, with the rapid growth in popularity of hedonistic-referenced affective accounts, we strongly recommend that future models in physical activity behavior account for these different hedonistic perspectives, and clearly define the version they refer to. While these inaccuracies may seem trivial, there may be important unintended consequences of misrepresenting hedonism in motivational theory. Primarily, we argue the main problem is that unclear definitions prevent academics from effectively challenging theoretical components. If authors argue that UPH underpins a particular theory, then key aspects of the UPH component can be contested. For instance, UPH is widely considered as unfalsifiable, which is empirically problematic for a theoretical model (Vroom, 1964; Locke, 1975; Steers et al., 2004). Moreover, serious logical corollaries may also be unnecessarily invoked. For instance, inadvertently adopting UPH rather than PPH implies an acknowledgment that human nature is purely deterministic, wherein humans have no free will to behave outside of their affective confines. Thus, the individual who passionately “gives themselves to the cause,” or behaves “out of principle,” would only be deemed to be altruistic or morally virtuous in terms of their effort to obtain the underlying pleasure or pain avoidance for them self alone. This approach would radically transform how many lay people view human nature, toward a self-centered or self-serving perspective (Nahmias et al., 2005; Baumeister, 2008; Sarkissian et al., 2010).

As Locke (1975) states, determinism “wipes out the possibility of ethics” (p. 462), leading UPH to be controversial at best, and morally repulsive at worst (Timmermann, 2005). On top of this, a deterministic view of human nature may also unnecessarily conflict with distal aspects of motivational theory, which are incompatible with determinism. For example, Self-Determination Theory (SDT) is inherently humanistic in its focus, with its emphasis on the growth of human potentials (Ryan et al., 2008). Yet, if SDT's hedonistic components are vague in definition, or if this theory overlooks different hedonistic perspectives, as is the case in one such article (Ryan et al., 2008), viewing SDT through a deterministic lens, in which individuals are inherently selfish, causes inherent conflict.

Mistakenly implicating UPH rather than PPH is likely to negatively impact the coherence of a theory or framework. Articles detailing Approach-Avoidance Motivation (AAM), for instance, often present hedonism as the intellectual basis of their framework (Elliot, 2006). However, Elliot (2006) also states: “Self-conceptions, implicit theories, attachment schemas, environmental affordance, cultural values and norms” (p. 114) can underpin goal-directed behavior. However he does this without explicitly delineating if such determinants, which would presumably be lower down in the hierarchy of needs, are simply a further means to obtain pleasure or reduce pain. Moreover, Higgins (1997) states hedonism to be “the basic motivational assumption of theories across all areas of psychology, including theories of emotion in psychobiology, conditioning in animal learning, decision making in cognitive and organizational psychology, consistency in social psychology, and achievement motivation in personality” (p. 1280). Yet Higgins (1997) appears to refer to PPH and not UPH, despite the fact Vroom (1964) and others (Locke, 1975; Steers et al., 2004) argue UPH is unfalsifiable and therefore empirically redundant.

Confronting this definitional issue is essential, and can be achieved within the physical activity literature by taking a few important steps. Firstly, making oneself aware of the different forms of hedonism that exist, and the consequential logical corollaries that may be implicated, is an essential precaution when operating in a field with inherent definitional challenges (Ekkekakis and Petruzzello, 2000). This increased awareness will promote the sensitivity in which hedonistic terms are used, and ensure the definitional problems that have occurred in other domains are prevented from developing. Secondly, if there is any chance of miss-representation of hedonistic terms, we advise authors to make explicit what they mean, rather than opting for brevity. Indeed, clearly discounting UPH in a text is preferable if there is potential for it to be implicated without intention.

To conclude, the future is promising for physical activity research. The field is moving away from models that exclusively account for social or cognitive components, and toward models that additionally account for affective components. However, the indiscriminant use of hedonistic terms in different areas of psychology and philosophy has hindered the ability to accurately determine which version of hedonism is actually intended. The aim of this article is to bring attention to the indiscriminate use of hedonistic terms, and highlight the consequential effects of such actions. It is hoped the physical activity literature will benefit from being better equipped, so as to use hedonistic terms more appropriately for the development of more refined theoretical models of behavior.

## Author contributions

SM conceived of the main argument and rationale for the paper during his Masters degree, under the supervision of DE. SM and DE developed and wrote the paper together and both authors approved revisions for journal submission.

### Conflict of interest statement

The authors declare that the research was conducted in the absence of any commercial or financial relationships that could be construed as a potential conflict of interest.
